# Understanding Intersectionality and Resiliency among Transgender Adolescents: Exploring Pathways among Peer Victimization, School Belonging, and Drug Use

**DOI:** 10.3390/ijerph15061289

**Published:** 2018-06-19

**Authors:** Tyler Hatchel, Robert Marx

**Affiliations:** 1 Department of Psychology, University of Florida, Gainesville, FL 36208, USA; 2 Department of Human & Organizational Development, Vanderbilt University, Nashville, TN 37235, USA; robert.a.marx@gmail.com

**Keywords:** school connectedness, substance abuse, LGBTQ youth, bullying

## Abstract

Transgender youth experience elevated levels of victimization and may therefore report greater drug use than their cisgender peers, yet little is known about protective factors like school belonging that may mediate this relationship. Further, scant research has explored the experiences of youth at the intersection of transgender identity and youth of color status or low socioeconomic status, especially with respect to these multiple minority statuses’ associations with peer victimization, drug use, and school belonging. Using data from the California Healthy Kids Survey, the current study employs structural equation modeling to explore the relationships among school belonging, peer victimization, and drug use for transgender youth. Findings indicate that school belonging does mediate the pathway between peer victimization and drug use for transgender youth and that although youth of color experience greater victimization, they do not engage in greater drug use than their white transgender peers. Based on these results, those concerned with the healthy futures of transgender youth should advocate for more open and affirming school climates that engender a sense of belonging and treat transgender youth with dignity and fairness.

## 1. Introduction

Gavin Grimm and Max Brennan, two Mid-Atlantic transgender high school students whose names have been in the news in the last three years, might seem to have much in common as they navigate adolescence, apply for college, and think about the future. However, Grimm, a student in Gloucester, VA, must use the bathroom that corresponds to the sex on his birth certificate, while Brennan and all other transgender students in Talbot County, MD, schools can use the bathroom that aligns with their gender identities [1,2]. Policies protecting transgender students in schools vary widely across the nation, either on a state-by-state or district-by-district basis, meaning that some students attend schools with policies that mandate administrators’ use of students’ correct names and pronouns, while others’ schools forbid it. This difference in policy may explain why in a recent national survey, 60% of transgender students reported being forced to use the restroom that corresponded to the sex on their birth certificate [3]. These policy differences likely have real consequences for students: in a national survey, 69.5% of transgender students reported avoiding bathrooms at school because they felt unsafe or uncomfortable, and students at schools without trans-supportive policies were more likely to report gender-related discrimination than their peers at schools with more supportive policies [3]. 

Gender discrimination likely has far-reaching consequences for transgender students in schools. Indeed, Grimm himself offers insight into the effects of unsupportive schools, saying, “When you’re singled out and sent a direct message to you and your peers that there’s something about you that needs to be segregated from the rest of the student body, it can add an extra level of stress and duress” [4]. Grimm’s observations are borne out in both the theoretical and empirical literature on transgender individuals. Drawing on the Minority Stress Model [5], scholars posit that transgender people face specific stressors related to their gender nonconformity, experiences with and expectations of victimization, and internalized transphobia [6]. Further, empirical research has demonstrated the increased stress and victimization transgender people face [7,8] and its association with increased depression [9,10], attempted suicide [11], drug use [12,13,14], and risky sexual behavior [12,15,16]. However, not all transgender students experience these poor outcomes, and perceived or actual social support may buffer against minority stress [17,18]. Supportive school climates can protect students from victimization and feeling unsafe [18,19], especially transgender youth of color (YOC) [20,21]. This understanding of transgender youth’s resilience, even in the face of stress, victimization, and negative outcomes, offers a more nuanced portrait of the ways transgender youth move through the world and cope with threats to their well-being. 

In line with previous scholarship that aimed to understand the relationships between victimization, negative behavioral outcomes, and the potential buffering effects of resilience, the current study investigates the role school belonging may play in mediating the relationship between peer victimization and drug use for transgender students. Further, the study explores the potential moderating effects of socioeconomic status (SES) and YOC status on the relationships among peer victimization, school belonging, and drug use.

### 1.1. Transgender Youth in Schools

The term transgender is used in this paper to describe individuals who understand or express their gender in ways that conflict with, enlarge upon, or transcend the social norms associated with their sex assigned at birth [22,23]. The term is intentionally broad, as transgender identities can take many forms and may be best conceived as an umbrella that includes those who move from one gender category to another, those who exist beyond the binary of gender, and those who move freely between and among genders [22]. It is beyond the scope of this paper to consider the multiplicity of forms transgender identities can take and the means by which some individuals socially or medically transition; rather, we employ the term to encompass all forms of gender expansiveness. Approximately 150,000 American adolescents, those aged 13 to 17, identify as transgender [24]. This is approximately 0.7% of the high-school-aged population in the United States. Importantly, these students may attend schools that are unsupportive or actively discriminatory against them; in a recent survey, 50.9% of transgender students reported that their school prohibited them from using their correct names and pronouns [25]. In addition to the structural policies that govern the names they can use, the bathrooms they can visit, and the sports they can play, transgender students also experience heightened peer victimization and stigma in school.

### 1.2. Peer Victimization

Peer victimization, the emotional, physical, or verbal abuse of a student by someone else in school, can take a variety of forms: peers can actively bully or harass with derogatory speech, physical violence, or behavior intended to do harm, or they can spread rumors, purposefully exclude from social groups, or intimidate [26,27,28,29]. For many transgender students, this peer victimization is a part of everyday life, so commonplace and ever-present that it shapes all aspects of their school experience. For example, in a recent study, 90% of transgender students reported hearing anti-LGBTQ slurs and negative comments about gender expression sometimes, often, or frequently in school [30]. Potentially more troubling, almost 40% of transgender students reported that school staff and personnel make negative comments about someone’s nonconforming gender expression [30]. In addition to this generalized and pervasive climate of transphobic comments, 87% of transgender students reported verbal harassment, 53% reported physical harassment, and 26% reported physical assault due to their gender identity and expression [30]. The National Transgender Discrimination Survey reported similar findings, noting that 78% of transgender individuals in K–12 schooling reported harassment, 35% reported physical assault, and 12% reported sexual assault [14]. 

In narrative reports, transgender students share similar experiences concerning their peers’ persistent demeaning, bullying, and threatening, especially for those youth whose gender expression is viewed as more nonconforming [15]. Additionally, transgender youth are often victimized not only for their gender identity, but also for their perceived sexuality; transgender students are often assumed to be non-heterosexual and therefore experience verbal and physical harassment due both to their gender expression and their perceived sexual orientation [30]. With such extreme victimization in schools, it is unsurprising that many transgender youth drop out of school, potentially to avoid the harassment they report experiencing [14,30]. It is clear that, for most transgender students, victimization is a part of daily life and shapes their experiences in schools.

### 1.3. Drug Use

Models of stress and coping often posit that increases in external stressors result in decreased capacity for rational decision-making and riskier behavior, including the use and abuse of drugs [5,31,32]. As marginalized individuals face increasing victimization, they may lose a sense of security, begin to devalue their own lives, and turn to maladaptive coping behaviors, such as the reliance on drugs and alcohol [5,33]. Transgender individuals, whose levels of victimization are higher than those in the general population, are at greater risk for drug abuse. A meta-analysis of 29 studies of transgender individuals found that 26.7% used illicit drugs, 43.7% abused alcohol, and 20.2% used cannabis, rates higher than those in the general population [34]. Among transgender youth, problems with substance abuse, recreational use of illicit substances, and reliance on substances as a coping mechanism were common [12,13,14]. In one study, 71% of young transgender women reported using cannabis and 65% reported using alcohol [12], and in another, 26% had used substances as a coping mechanism to attempt to manage discrimination and victimization [14]. In an analysis of transgender girls in San Francisco, 69% of the youth reported using substances within the previous six months, and those with post-traumatic stress disorder and those who experienced discrimination based on their gender expression were significantly more likely to use drugs [35]. When examining stressors within schools, transgender students who reported greater victimization in school also reported higher rates of drug abuse, and those who reported physical assault reported twice the rate of drug abuse [14]. 

### 1.4. Resilience and School Belonging 

Resilience is a complicated construct and has therefore been conceptualized in divergent fashions. Experts in the field define resiliency broadly as the capacity to overcome, and adapt in the face of, adversity [36]. Part of the discourse has been concerned with whether resiliency should be conceptualized as a static characteristic or a dynamic and developmental process. The present paper will conceptualize resiliency as a dynamic process and will focus on related factors such as stigma based on group membership and peer relations. More specifically within a school context, individual risk factors are conceptualized as the prevalence of peer victimization as a function of diverse group membership and perceptions of belonging and school-based support as protective factors. This draws on a multidimensional conception of resiliency, rather than the notion that resiliency is a single trait, as we recognize that exposure to adversity is not necessary for resilience and that a more multifaceted understanding of resilience is useful for intervention [37]. This has been substantiated by findings that demonstrate how resiliency can manifest in differentiating domains or emerge in response to particular forms of adversity [37].

Although transgender youth experience high levels of victimization and drug use, their school lives are not monolithic and uniform. Instead, several protective factors may serve to buffer students from the negative effects of victimization and foster resilience. For example, transgender youth’s perceptions that they are socially supported (i.e., that they can count on their family and friends) were associated with positive mental health outcomes [18]. Within schools, transgender youth highlighted the importance of supportive educational systems, especially school counselors and other staff who were knowledgeable about transgender issues, as a source of resilience that enabled them to successfully manage the stressors they faced [21]. Students also referenced the importance of their abilities to advocate for themselves within the school environment, whether as a member of an organization focused on transgender issues or as a student in a class advocating for the use of their correct name and pronouns [20].

Schools can also actively work to foster more supportive environments for transgender youth so that the stressors they face are mitigated and managed. For example, schools in which teachers intervene when they hear gender-discriminatory remarks, schools in which LGBTQ topics are incorporated into the curricula, schools with organizations for LGBTQ youth, and schools with knowledgeable and informed personnel are associated with greater feelings of connection to school staff and feelings of safety [8]. Additionally, supportive personnel, LGBTQ-inclusive curricula, and organizations for LGBTQ youth were associated with lower rates of absenteeism and victimization among transgender students [19]. 

These findings about facets of schools that are associated with transgender student resilience and managing of stressors align with the broader literature on school belonging, the extent to which students view their school as a community that meets their need for inclusion and acceptance [38]. Schools that are seen as welcoming, supportive environments foster adolescent development along multiple dimensions and enable successful identity formation [38], and reduce the likelihood of risky or maladaptive behavior [39,40]. Importantly, greater school belonging is associated with decreased rates of drug use, as eighth grade students who reported high school belonging were less likely to smoke cigarettes, drink alcohol, or use cannabis following graduation from high school [41].

Unfortunately, many transgender students report low levels of school belonging, and on average, report lower levels of belonging than even their LGB peers [30]. This may stand to reason, as they also report greater victimization than their LGB peers and are more likely to experience harassment and assault [25]. Just as with their LGB peers, though, certain school-based supports are associated with greater feelings of school belonging: transgender students who discussed their own gender expression or sexual orientation and who discussed LGBT topics in schools were more likely to report a sense of belonging to their school [30]. Dishearteningly, many transgender students reported that they did not have resources associated with more supportive school environments: less than half of the students reported the presence of an LGBTQ club at school or the ability to find information about LGBTQ people or history [30]. It seems, then, that although indicators of supportive school environments are known, schools have been slow in the uptake of such resources, and therefore transgender students tend to feel low levels of school belonging. 

### 1.5. Youth of Color Status and Socioeconomic Status

Transgender youth are not a monolithic group. Their experiences are marked by many aspects of their identities, and in attempting to better understand their lives, researchers should attend to the intersecting forces that combine to create specific oppression and marginalization [42,43]. Two salient identity areas that should be further explored are SES and YOC status. Research on LGBTQ YOC demonstrates that they are often more likely to experience victimization and harassment in school [25,44,45], and transgender YOC experience higher rates of harassment and violence than their peers [14]. Nonetheless, research has identified no relationship between transgender YOC status and drug use [46,47]. Qualitative research indicates that transgender YOC are often resilient in the face of racism and transgender prejudice, self-authoring strong racial and gender identities, advocating for themselves, and developing communities [20]. These findings align well with research on all YOC. Johnston and colleagues offer a report on about 43,700 students in 360 schools and found that the prevalence of drug use was higher among white youth when compared to ethnically diverse peers [48]. 

Research on SES is markedly more limited. Consistent with findings in the general population, high family SES is associated with better general health and fewer depressive symptoms for LGBTQ young adults [49], and high income is associated with resilience for transgender adults [50]. However, researchers have not investigated the associations between SES and victimization, school belonging, or drug use for queer youth. In sum, though, the research on YOC status and SES is mixed and inconclusive, especially as it relates to transgender youth’s experiences.

### 1.6. The Current Study

Previous research has established that transgender youth experience high levels of victimization, are more likely to use drugs, and feel low levels of school belonging. The purpose of this study was to investigate the relationships among peer victimization, school belonging, and substance use among high-school-aged transgender youth. The study aims to answer the following research questions:

1. Do SES and YOC status moderate the relationship between peer victimization and drug use for transgender youth?
2. Does school belonging mediate the relationship between peer victimization and drug use for transgender youth?
3. Do SES and YOC status moderate the mediation of school belonging?

## 2. Materials and Methods

### 2.1. Study Design, Participants and Procedures

The present paper analyzed a subsample of cross-sectional data from the 2013–2015 California Healthy Kids Survey (CHKS). The CHKS is a state-wide, biannual assessment of school climate and student health in the State of California among middle and high school students [51]. The full dataset offered information on 634,978 participants. The sub-sample comprised 4778 youth who identified as transgender. Inconsistent participant responses like reporting never using a drug in their lifetime and then reporting using a drug in the past thirty days, reporting using a fake drug, or self-reporting dishonest responses were the criteria used to exclude participants from the final sample [52]. The following exclusions—disparities between drug use reports, n = 57; reported using a fake drug, n = 0; self-reported being dishonest, n = 0—resulted in a total of 4721 transgender participants comprising the final sample.

Demographic information included self-reports of sex (female = 40.5%; male = 55.3%, missing = 4.2%), age (*M* = 14.71 years; range = 10–18 years), ethnicity (American Indian or Alaska Native = 4.6%, Asian = 10.3%, Black or African American = 7.3%, Native Hawaiian or Pacific Islander = 2.6%, Mixed = 36.1%, White = 29.9%, and missing = 9.1%), and whether they were of Hispanic or Latin origin (yes = 44.9%, no = 51.9%, missing = 3.2%). 

The California Healthy Kids Survey was developed by WestEd for the California Department of Education [52]. District level, school level, and state level approvals were obtained. Active parent consent was obtained for students in grades below 7 and passive consent was obtained for students in grades 7 to 11. Parents were notified of their right to inspect the CHKS, and procedures were established to accommodate their access to the survey [52]. The survey was administered by teachers or by trained staff from WestEd. Participants could withdraw from the survey at any time. A Memorandum of Understanding including a Confidentiality Agreement was signed by authors who have access to the data. The current study employed secondary analyses of an existing publicly available de-identified dataset provided by the California Department of Education. Therefore, this study did not require oversight or review by the Institutional Review Board.

### 2.2. Measures

**Peer Victimization.** One survey measured violence, safety, harassment and bullying on school property. Only items measuring peer victimization explicitly, not items on violence and school safety, were included. Participants were asked, “During the past 12 months, how many times on school property have you…”—“been pushed, shoved, slapped, hit, or kicked by someone who wasn’t just kidding around?”, “been afraid of being beaten up”, “had mean rumors or lies spread about you”, “had sexual jokes, comments, or gestures made to you”, “been made fun of because of your looks or the way you talk?”, “had your property stolen or deliberately damaged, such as your car, clothing, or books”, “been threatened or injured with a weapon (gun, knife, club, etc.)?”, “been threatened with harm or injury”, and “been made fun of, insulted, or called names”. Response options included “0 time”, “1 time”, “2 to 3 times”, and “4 or more times.” The Cronbach’s Alpha coefficient was 0.91. A latent variable was used to account for measurement error.

**Drug Use.** Drug use was measured by 10 items exploring a variety of substances. Participants were asked “During the past 30 days, on how many days did you use…”—“cigarettes?”, “smokeless tobacco (dip, chew, or snuff)?”, “electronic cigarettes, e-cigarettes, or other vaping device such as e-hookah, hookah pens, or vape pens?”, “one or more drinks of alcohol?”, “five or more drinks of alcohol?”, “marijuana (smoke, vape, eat, or drink)?”, “inhalants (things you sniff, huff, or breath to get “high”)?”, “prescription drugs to get ‘high’ off of for reasons other than prescribed?”, “any other drug, pill, or medicine to get ‘high’ or for reasons other than medical?”, and “two or more substances at the same time (for example, alcohol with marijuana, ecstasy with mushrooms)?” Response options were “0 day”, “1 day”, “2 days”, “3–9 days”, “10–19 days”, and “20–30 days”. The Cronbach’s Alpha was 0.93. A latent variable named drug use was employed to adjust for measurement error. 

**Transgender Identity.** A one-item question asked participants about their gender identity. The questioned offered some background and a question, “Some people describe themselves as transgender when their sex at birth does not match the way they think or feel about their gender. Are you transgender?” Response options included “No, I am not transgender”, “Yes, I am transgender”, “I am not sure if I am transgender”, and “Decline to respond.” Only participants who reported that they were transgender were included in this study.

#### 2.2.1. Mediators

**School Belonging.** School belonging was measured via five items. Participants were asked “How strongly do you agree or disagree with the following statements?”—“I feel close to people at this school”, “I am happy to be at this school”, “I feel like I am part of this school”, “The teachers at this school treat students fairly”, and “I feel safe in my school.” Response options were “strongly disagree”, “disagree”, “neither disagree nor agree”, “agree” and “strongly agree.” The Cronbach’s Alpha was 0.85. A latent variable, school belonging, was utilized to adjust for measurement error.

#### 2.2.2. Moderators

**Socioeconomic Status.** One question was used to represent SES. Participants were asked, “What is the highest level of education your parents or guardians completed? (Mark the educational level of the parent or guardian who went the furthest in school)?” Response options included, “did not finish high school”, “graduated from high school”, “attended college but did not complete a four-year degree”, “graduated from college”, and “don’t know”. This variable was dichotomized such that students who reported that their parents did not complete high school had a low SES when compared to their peers. 

**Youth of Color Status.** Two questions were utilized to classify students as people of color—“Are you of Hispanic or Latino origin?” and “What is your race?”. This factor was dichotomized such that students who did not report being white were coded as YOC. 

### 2.3. Overview of Analysis

The assumptions of normality were satisfactory for the variables. SPSS was utilized to run the descriptive analyses, and AMOS (IBM, Inc., Chicago, IL, USA) was used to complete modeling. 

Structural equation modeling (SEM) was employed to estimate relations among peer victimization, school belonging, and drug use among transgender youth. Multi-group confirmatory factor analyses (CFA) were used to examine latent differences and invariance [53]. Model fit was determined by chi-square test (χ^2^), root mean square error of approximation (RMSEA), comparative fit index (CFI), and non-normed fit index (NFI). A significant χ^2^ could indicate poor model fit, but this can be a biased estimate when larger samples are used [54]. The covariates age and ethnicity were controlled for in the mediation analysis. 

Invariance testing was conducted utilizing a multi-group CFA framework with the objective of examining measurement invariance across transgender youth with different SES and YOC statuses. The effects coding method was employed to test for invariance on three levels—configural invariance (i.e., the pattern of fixed and free parameters is the same), weak factorial invariance (i.e., loadings have the same value in each group), and strong factorial invariance (i.e., measured intercepts have the same value in each group) [55]. Little’s “reasonableness” tests were utilized to show invariance by indicating that the change in CFI ≤ 0.01 and that the RMSEA fits in the previous model’s RMSEA confidence interval (CI) [56].

Three levels were also examined for initial structural models—factor mean invariance (i.e., all factor means are the same across groups), factor variance invariance (i.e., all factor variances are the same across groups), and factor covariance invariance (i.e., all factor covariances are the same across groups). This analysis conducts an examination of moderation by comparing groups. To examine if group differences moderated the mediation model, regression weights were compared as opposed to factor covariances. 

Parcels were used given their benefits, including improved reliability, more communality, and reduced violations of distributions assumptions [57]. An item-to-construct balance method was utilized for all three of the latent constructs of interest—peer victimization, drug use, and school belonging [57]. An exploratory factor analysis was conducted via maximum likelihood estimation, one fixed factor, and a promax rotation. The factors were divided into three parcels for each latent construct. The items were averaged as opposed to summed so that the original scales were preserved. 

### 2.4. Missing Data

Missing data ranged from 0.03–4.2%. Little’s missing completely at random test was significant, indicating that there was possibly a pattern in missing data (χ^2^ = 17.11, *df* = 7, *p* = 0.017). However, it is considered challenging to authenticate whether the data are missing at random (MAR) or missing not at random. Schafer and Graham explain that MAR can only be strictly confirmed by procurement of follow-up data or by contributing an unverifiable model [58]. 

Missing approximately 4% or less data is considered reasonable. Furthermore, missing analyses suggest that possible patterns were associated with demographic variables such as age, YOC status, and SES. Including these auxiliary variables (i.e., as covariates) in the model should protect potential biases in parameter estimation. Full Information Maximum Likelihood (FIML) estimation was also utilized to handle missing data since it is a powerful approach when compared to other options [53]. FIML uses all of the accessible data and offers estimates that account for potential biases that exist in the data.

## 3. Results

### 3.1. Descriptive Statistics

Table 1 offers a summary of means, standard deviations, and bivariate correlations of the manifest variables. Seventy-two percent of transgender youth reported being victimized by their peers in the past 12 months. Thirty-six percent of the sample shared that they used at least one substance in the past 30 days. Fifteen percent of transgender youth were coded as low SES. Seventy-four percent of transgender youth were classified as being YOC.

### 3.2. Measurement Models 1 and 2

Invariance testing explored potential group differences across the specified parameters (i.e., SES and YOC status). Findings indicated that strong factorial invariance held for peer victimization and drug use. Therefore, the two constructs were measured consistently across all four groups (see Supplementary Materials).

## 4. Moderation

### 4.1. Structural Model 1

Invariance testing was used to observe structural invariance across transgender youth with different SES. Findings demonstrated that the groups were invariant with regards to variance and covariance, but varied as a function of means. This suggests that SES did moderate the relationship between peer victimization and drug use. Cohen’s *d* effect sizes for the mean differences on each latent construct were calculated. Transgender youth of low SES (*M* = 0) reported more victimization than transgender youth not of low SES (*M* = −0.12), indicating approximately a fifth of a standard deviation more victimization (*d* = 0.17). Likewise, transgender youth of low SES (*M* = 0) reported more drug use than transgender youth not of low SES (*M* = −0.27), indicating approximately two-fifths of a standard deviation more drug use (*d* = 0.38). These mean differences suggest that the transgender youth of low SES were exposed to more victimization and engaged in further drug use than their transgender peers without low SES (see Supplementary Materials).

### 4.2. Structural Model 2

Invariance testing was utilized to examine structural invariance across YOC statuses. Results showed that the groups were invariant. Thus YOC status did not moderate the relationship between peer victimization and drug use (see Supplementary Materials). 

All of the groups were combined after invariance testing and the structural model exploring the relation between peer victimization and drug use was tested. The analysis confirmed good model fit, χ^2^(8, *n* = 4778) = 104.75, *p* < 0.01, RMSEA = 0.05 [0.042, 0.059], NFI = 0.996, CFI = 0.996.

## 5. Mediation

### 5.1. Measurement Models 3 and 4

Invariance testing analyzed potential measurement disparities across all of the groups of transgender youth (e.g., SES and YOC status). Findings demonstrated that strong factorial invariance held for school belonging in addition to the other constructs (see Table 2 and Table 3). 

### 5.2. Structural Model 3

**Figure 1:**
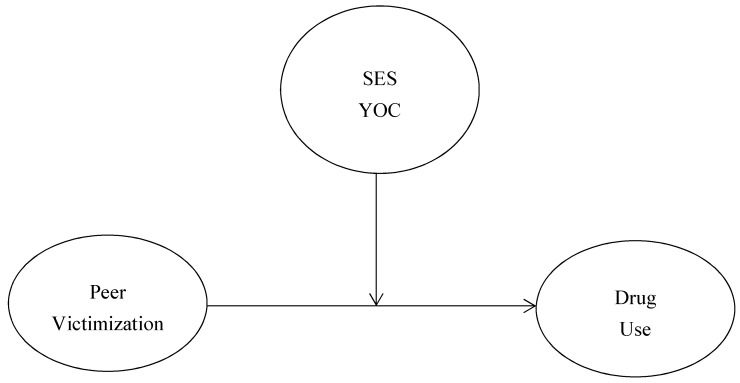
Conceptual Moderation Models. *Note.* SES = socioeconomic status, YOC = youth of color status.

**Figure 2:**
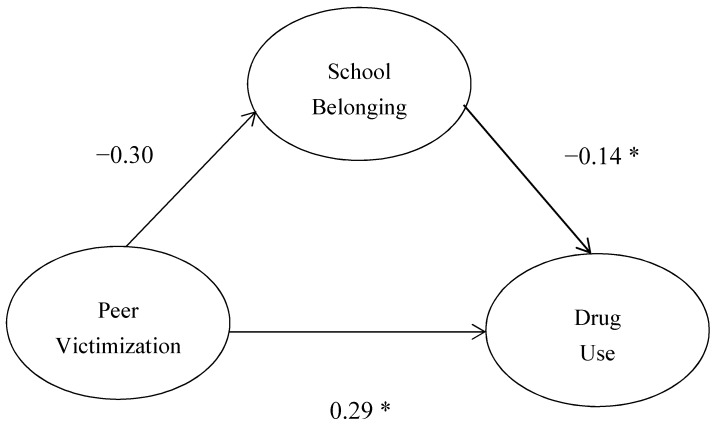
Structural equation model with latent variables and standardized estimates; * = *p* < 0.001.

This structural model examined whether school belonging mediates the relationships between peer victimization and drug use for the whole sample. Analyses showed that peer victimization had a direct effect on drug use (b = 0.29, *p* < 0.001) and school belonging (b = −0.29, *p* < 0.001). School belonging had a direct effect on drug use (b = −0.14, *p* < 0.001) (Figure 1). The model demonstrated good fit, χ^2^(17, *n* = 4778) = 137.53, *p* < 0.001, RMSEA = 0.04 (0.033, 0.045), NFI = 0.995, CFI = 0.994 (Figure 2). Peer victimization and school belonging accounted for 22% of the variance in drug use. 

RMediation was used to estimate the indirect effect of peer victimization, through school belonging, on drug use. This technique utilizes a distribution-of-product method to evaluate significance levels of the indirect effects by estimating a 95% CI [59]. The indirect effect is considered significant if the CI does not include zero in the range. The indirect effect of peer victimization through school belonging on drug use was significant (b = 0.05, *SE* = 0.007, 95% CI [0.036, 0.062]).

## 6. Moderated Mediation

### 6.1. Structural Model 4

Invariance testing was utilized to examine whether group differences emerged in the mediation model. Model 5 compared transgender youth who were low SES versus those who were not. Fit indices showed that the groups did not vary in regression coefficients, but did vary in means (Table 2). These findings suggest that mean differences exist (see structural model 1), but that SES did not moderate indirect effects in the mediation model. 

### 6.2. Structural Model 5

This model analyzed possible group differences as a function of YOC status. Results via the reasonable test suggested that the groups were invariant in means, variances and regression coefficients (Table 3). Findings demonstrated that YOC status does not moderate the mediation model.

## 7. Discussion

The goal of this study was to explore relationships among peer victimization, school belonging, and drug use among transgender youth as well as the role of their intersectional identities. There is a clear dearth of research that examines these factors among transgender youth and, therefore, this study offers a unique contribution to the extant literature [60].

Descriptive findings illustrated that a disconcerting number of transgender youth were subjected to peer victimization and engaged in drug use. These findings are consistent with other reports which demonstrated that transgender youth are exposed to frequent peer victimization [14,30]. Research has also shown that marginalized people like transgender youth may turn to drugs to deal with factors like peer victimization and stigma [3,13,14,35,46]. 

An examination of the moderation of the relationship between peer victimization and drug use offered important insights. Findings demonstrated that although transgender youth with low SES were at risk for more victimization and therefore more drug use, youth with YOC status were not. This indicates that although both transgender youth of low SES and transgender YOC experienced greater victimization than their high SES and white transgender peers, only transgender youth of low SES in turn reported greater drug use. Transgender YOC did not report greater drug use than their white transgender peers, despite experiencing more victimization. The existing literature documents mixed findings in terms of the intersectionality of multiple marginalized identities. Some have established that YOC may be targeted by more victimization than their peers [25,44,45], in line with our findings. Other studies demonstrate that transgender YOC status does not predict drug use [46,47], also in line with our results. Findings have also suggested that intersecting identities can be conceptualized as a feature of resiliency [20,61,62] and that YOC were less likely to use drugs than their white peers [48]. Our data align with these frameworks and findings, demonstrating that YOC were not at additional risk for drug use, despite the increased victimization they reported.

A sense of school belonging is essential for most youth, and it is likely even more important for stigmatized youth. Our mediation analysis demonstrated that peer victimization predicted diminished school belonging and that a greater sense of school belonging was associated with less drug use. Although increased peer victimization is associated with increased drug use, school belonging appears to be an essential psychological component than can illuminate the connection between stigmatization and poor outcomes. As some research posits that victimized youth turn to drugs as a coping mechanism, our findings demonstrate that youth may experience the protective value of a supportive school community and, in turn, not use drugs. These findings align well with the existing research that established perceptions of belonging and support as key dimensions of resiliency [8,18,19,21]. Exploring group differences in these data demonstrated that having low SES or YOC status was not key to the role of school belonging. That is, all transgender students would benefit equally from an increased sense of acceptance and belonging.

## 8. Limitations

Despite the innovative contributions of this paper, there are limitations that must be acknowledged. The sampling methodology only included one geographical location, limiting the generalizability of the results. This is especially true given the unique characteristics of California, an ethnically diverse state with more progressive school policies. It is reasonable to speculate that transgender youth across the nation—especially YOC and low SES transgender youth—may feel more stigmatized and excluded than youth in California. Additionally, the study was limited by the confines of the measurement and design of the primary data. The measurement of SES was based on only one item, although best practice considers SES a complex construct that benefits from a more comprehensive exploration. Similarly, the measurement of transgender status was not in line with the ideal two-step process which asks about sex assigned at birth as well as current gender identity, nor was specific gender identities measured [63]. In order to understand differences along YOC status, we dichotomized into YOC and white transgender youth and did not illustrate differences that exist within the YOC category. Our models, in turn, addressed low SES and YOC status individually (i.e., focused on low SES transgender youth *or* transgender YOC) rather than considering them together (i.e., focusing on transgender youth who were both low SES and YOC). Finally, the present paper utilized a cross-sectional design and reports from a broad age range of participants. As such, the paper does not address developmental periods or directionality.

## 9. Conclusions

In the hopes of building on the current study, future research should draw from the strengths of this study while potentially mitigating some of its limitations. Research that employs a nationally representative sample and that could offer a broader understanding of the relationships among peer victimization, school belonging, and drug use will extend these findings considerably. Further, research that could provide important contextual information about the schools, communities or regions that are most successful in cultivating a sense of school belonging for transgender youth will have important implications for educational policy. Indeed, future research should aim to offer both a comprehensive view of school belonging’s role in the relationship between peer victimization and drug use and a local, granular understanding of the differences in that role across school contexts, settings and locations. 

As the current study provides promising evidence that school belonging may be an important factor for decreasing drug use among transgender youth, school administrators should work to create open and affirming school climates that encourage transgender youth—and, indeed, all youth—to form strong connections, to feel safe and welcome, and to be their authentic selves. The push for increased school belonging could take many forms, including the enactment of policies that ensure that transgender students can use their correct names and pronouns, the creation and support of clubs like Gender and Sexuality Alliances, and the training of school personnel in working with all students. The current study also makes clear the high rates of peer victimization that transgender students, and especially transgender YOC, experience at school. With that in mind, school administrators should examine the intersectional oppression that may shape the lives of transgender YOC, working to combat both heterosexism and racism in their schools and considering the ways in which power operates within the school context to disadvantage transgender youth in general and transgender YOC specifically. Finally, this study contributes to the growing body of literature on transgender YOC resilience that serves to demonstrate the potential protective factors of multiple minority statuses. The present research helps to reframe the narrative surrounding vulnerable populations by acknowledging the complexities that shape young people’s lives.

Drug use is a widespread public health concern, and this research offers a new and fruitful way of understanding a potential countervailing force that could be leveraged to improve youth outcomes. Creating open and affirming school climates where transgender youth feel a sense of belonging is one important strategy for limiting youth’s drug abuse and ensuring healthy futures for all, especially those who may experience multiple sources of stress and oppression as they live their authentic lives. The value of improving the well-being of LGBTQ adults and youth via affirmative interventions is established [64,65]. Therapy models utilize affirmation as a tool to disrupt the stigma to poor outcomes pathways. It follows that affirmative school-based interventions are clearly better approaches to supporting these youths. Unfortunately, there are often barriers to incorporating affirmative interventions in schools—including stigma within the community as well as policies at varying systematic levels (i.e., federal, state, district, school). The dismantling of these social and systematic barriers will facilitate the well-being of transgender youth.

## Figures and Tables

**Table 1:** Manifest Scale Bivariate Correlations, Means and Standard Deviations.

Measures	*M*	*SD*	Peer Victimization	Drug Use
Peer Victimization	1.81	0.87	-	
Drug Use	1.47	0.98	0.30	-
School Belonging	3.16	1.01	−0.26	−0.22

*Note*. All correlations significant at *p* < 0.01.

**Table 2:** Fit Indices for Multigroup Invariance Comparisons Based on SES—Measurement model 3 and SEM model 4.

Model	χ^2^	*df*	*p*	RMSEA	RMSEA 95% CI	NFI	CFI	ΔCFI	Pass?
Measurement Invariance									
Configural	146.08	34	<0.05	0.03	(0.022, 0.031)	0.994	0.996		Yes
Weak	151.13	39	<0.05	0.03	(0.021, 0.030)	0.994	0.996	0.000	Yes
Strong/Scalar	187.78	44	<0.05	0.03	(0.023, 0.031)	0.993	0.994	0.002	Yes
Structural Invariance									
Factor Means	372.24	55	<0.05	0.04	(0.032, 0.034)	0.986	0.988	0.006	No
Factor Variances	480.28	58	<0.05	0.04	(0.036, 0.043)	0.981	0.984	0.004	Yes
Equal Regression	545.94	61	<0.05	0.04	(0.038, 0.045)	0.979	0.981	0.003	Yes
Unequal Regression	381.10	56	<0.05	0.04	(0.032, 0.039)	0.985	0.987	0.006	Yes

*Note.* Δ = the change in value compared to previous model; RMSEA = root mean square error of approximation; CI = confidence interval; NFI = non-normed fit index; CFI = comparative fit index, SES = socioeconomic status. Pass evaluated by ΔCFI ≤ 0.01 and RMSEA falling in the previous model’s RMSEA CI.

**Table 3:** Fit Indices for Multigroup Invariance Comparisons based on YOC Status—Measurement model 4 and SEM model 5.

Model	χ^2^	*df*	*p*	RMSEA	RMSEA 95% CI	NFI	CFI	ΔCFI	Pass?
Measurement Invariance									
Configural	144.93	34	<0.05	0.03	(0.022, 0.031)	0.994	0.996		Yes
Weak	166.63	39	<0.05	0.03	(0.022, 0.031)	0.994	0.995	0.001	Yes
Strong/Scalar	187.55	44	<0.05	0.03	(0.023, 0.030)	0.993	0.994	0.001	Yes
Structural Invariance									
Factor Means	258.84	55	<0.05	0.03	(0.025, 0.032)	0.990	0.992	0.002	Yes
Factor Variances	287.26	58	<0.05	0.03	(0.026, 0.033)	0.989	0.991	0.001	Yes
Equal Regression	293.55	61	<0.05	0.03	(0.025, 0.032)	0.989	0.991	0.000	Yes
Unequal Regression	260.56	56	<0.05	0.03	(0.028, 0.032)	0.990	0.992	0.001	Yes

*Note.* Δ = the change in value compared to previous model; RMSEA = root mean square error of approximation; CI = confidence interval; NFI = non-normed fit index; CFI = comparative fit index, YOC = youth of color. Pass evaluated by ΔCFI ≤ 0.01 and RMSEA falling in the previous model’s RMSEA CI.

## Supplementary Materials

The following are available online at http://www.mdpi.com/1660-4601/15/6/1289/s1, Table S1: Fit Indices for Multigroup Invariance Comparisons Based on SES—Model 1. Table S2: Fit Indices for Multigroup Invariance Comparisons based on POC Status—Model 2.
