# Supplementary material for: Understanding Intersectionality and Resiliency among Transgender Adolescents: Exploring Pathways among Peer Victimization, School Belonging, and Drug Use
Source: Int J Environ Res Public Health. 2018 Jun 19;15(6):1289. doi: 10.3390/ijerph15061289 (PMC6025184; doi:10.3390/ijerph15061289)
Supplement: Supplementary file 1 [file ijerph-15-01289-s001.pdf]

Table S1. Fit Indices for Multigroup Invariance Comparisons Based on SES – Model 1

Model	χ^2	*df*	*p*	RMSEA	RMSEA 90% CI	NFI	CFI	ΔCFI	Pass?
				Measurement Invariance					
Configural	107.15	16	<0.05	0.04	[0.029, 0.041]	0.995	0.996		Yes
Weak	181.95	20	<0.05	0.04	[0.036, 0.047]	0.992	0.993	0.003	Yes
Strong/Scalar	192.68	24	<0.05	0.04	[0.034, 0.044]	0.992	0.993	0.000	Yes
				Structural Invariance					
Factor Means	389.38	32	<0.05	0.05	[0.045, 0.054]	0.983	0.984	0.009	No
Factor Variances	494.64	34	<0.05	0.05	[0.050, 0.058]	0.978	0.980	0.004	Yes
Factor Covariance	497.28	35	<0.05	0.05	[0.049, 0.058]	0.978	0.980	0.000	Yes

Note. Δ = the change in value compared to previous model; RMSEA = root mean square error of approximation; CI = confidence interval; NFI = non-normed fit index; CFI = comparative fit index, SES = socioeconomic status. Pass evaluated by ΔCFI ≤ 0.01 and RMSEA falling in the previous model's RMSEA CI.

Table S2. Fit Indices for Multigroup Invariance Comparisons based on POC Status – Model 2

Model	χ^2	*df*	*p*	RMSEA	RMSEA 90% CI	NFI	CFI	ΔCFI	Pass?
				Measurement Invariance					
Configural	105.04	16	<0.05	0.03	[0.028, 0.041]	0.995	0.996		Yes
Weak	125.66	20	<0.05	0.03	[0.028, 0.039]	0.995	0.995	0.001	Yes
Strong/Scalar	137.41	24	<0.05	0.03	[0.027, 0.036]	0.994	0.995	0.000	Yes
				Structural Invariance					
Factor Means	170.39	32	<0.05	0.03	[0.026, 0.035]	0.993	0.994	0.001	Yes
Factor Variances	186.85	34	<0.05	0.03	[0.027, 0.036]	0.992	0.993	0.001	Yes
Factor Covariance	192.01	35	<0.05	0.03	[0.027, 0.035]	0.992	0.993	0.000	Yes

Note. Δ = the change in value compared to previous model; RMSEA = root mean square error of approximation; CI = confidence interval; NFI = non-normed fit index; CFI = comparative fit index, POC = person of color. Pass evaluated by ΔCFI ≤ .01 and RMSEA falling in the previous model's RMSEA CI.
